# Miss Manson's Paper: Two Protests

**Published:** 1887-04-23

**Authors:** 


### MISS MANSON'S PAPER—TWO PROTESTS.

By Miss M. Mollett, Lady Superintendent, National Hospital for the Paralysed and Epileptic, Queen Square, Bloomsbury.

I should be much obliged if you would allow me a short space in your journal to protest against the tone which has been allowed to appear in the discussion provoked by Miss Manson's paper on Private Nursing Institutions. The articles which have appeared in The Hospital in answer to Miss Manson's paper have struck me as being, with a few marked exceptions, curiously illogical and beside the main question. I must say that I have been exceedingly surprised, in reading some of them over, with the personal nature of the arguments used, and the want of any desire to conform with the customary rules of literary courtesy that they display.

Miss Manson attacked a principle which she believed, rightly or wrongly, to be wrong,—and which anyone was, of course, at perfect liberty to attack or defend ; it never, I am certain, entered her mind to contradict, what is as patent to her as to every sensible woman, the fact that some private institutions are well managed, any more than it would surely enter the mind of anyone to deny that many abuses exist in connection with Private Nursing Institutions. Instead of denying the justice of her attack on a general principle, the superintendents and private nurses who have rushed into print on this occasion, have principally confined themselves to defending the arrangements of their own private institutions, or abusing hospital private nursing arrangements generally. No one denies that there are well managed Private Homes. No one denies that abuses exist in hospitals. One part of Miss Manson's paper was devoted to expressing her most firm opinion that the private nursing of a hospital should be separated from the ward nursing, which principle is most rigorously enforced in the Private Nursing Home attached to St. Bartholomew's ; nevertheless, letters in answer to her paper continually reiterate, as one of the main arguments in favour of the present system of Private Nursing Institutions, the harm done to hospital patients by the removing of nurses from the wards to take charge of private patients, or of the harm done to private patients by sending raw, half-trained nurses straight from hospital wards. That, however, is merely a detail,—but when the ladies who reply to Miss Manson consider it necessary to drag in matters connected with her private life, irrelevant wholly to the matter in hand, I think it is decidedly time for some one to protest, in the name of all gentlewomen connected with hospital work, against continuing a discussion which, if carried on in such a strain, can hardly be productive of much benefit to hospital reform. It is not encouraging to those who would like to give the hospital world the benefit of their experience in subjects in which they are interested, to know that they are laying themselves open to a personal discussion of their private affairs, which is surely unnecessary in a public paper.

By the Lady Superintendent of a Provincial Nursing Institution.

I have read your remarks in to-day's (16th inst.) Hospital, and hardly know what construction to put upon them; but I do think, if the word "abuse" is used, it ought to be in connection with what Miss Manson has said about Private Nursing Institutions, of which she has evidently no knowledge. At least, if there are one or two that are not conducted properly, that is no reason for condemning the whole. As well condemn all hospitals because *one* is not conducted properly. As well as being Lady Superintendent here for four years, I have held every post in hospitals, even to Lady Superintendent *pro tem.*, so I ought to know something of what I write about. I can assure Miss Manson, or anyone, that, so far as my knowledge goes, we have to send out the very best nurses, very superior to what I have seen sent from hospitals ; but I must decline any further correspondence if it is to lay me open to such remarks as "descending to the level of personal abuse." In sending my letters, I gave you the option of publishing, and if you thought them objectionable, I should have expected you to suppress them. I could certainly not be an enemy, as you suggest, to Miss Manson, as I never saw her, and did not even know where she hailed from when first I wrote. This is simply written so that you may know why I do not write or take any interest in the subject in future, as I shall not lay myself open to such remarks again. I can equally claim with your correspondents to express the views of many other ladies, and it would be the last thing that anyone would give me credit for to give personal abuse.

### A NEW DEPARTURE.

On Wednesday evening last The Hospitals Association took a new departure by holding their fifth general meeting at one of the hospitals, the Brompton Hospital for Consumption, which is certainly a step in the right direction. "The Ventilation of Hospitals" was an excellent subject for the paper, and Dr. Williams was able to illustrate many practical points in the course of the inspection of the splendid new buildings, after the meeting. As might have been expected, the audience was unusually representative and influential, and the whole of the arrangements reflect the greatest credit upon the representatives of the Brompton Hospital. We venture to express the hope that during each session, at least every alternate meeting of The Hospitals Association will in future be held at some of the hospitals. Dr. Williams' instructive and interesting paper will be found in full in another part of this issue.

